# Systematic suppression of *Leishmania* (*Leishmania*)* amazonensis*-mediated delayed-type hypersensitivity response in American cutaneous leishmaniasis

**DOI:** 10.1186/s13071-025-06941-6

**Published:** 2025-08-05

**Authors:** Marliane B. Campos, Luciana V. R. Lima, Thiago Vasconcelos dos Santos, Patrícia K. Ramos, Claudia M. C. Gomes, Márcia D. Laurenti, Carlos E. P. Corbett, Jeffrey J. Shaw, Fernando T. Silveira

**Affiliations:** 1 Evandro Chagas Institute (Surveillance Secretary of Health and Environment, Ministry of Health), Ananindeua, Pará Brazil; 2 Medical School of São Paulo University, São Paulo, São Paulo Brazil; 3 Biomedical Sciences Institute, São Paulo University, São Paulo, São Paulo Brazil; 4 Tropical Medicine Nucleus, Federal University of Pará, Belém, Pará Brazil

**Keywords:** *Leishmania* (*L.*) *amazonensis*, Systematic suppression, T-cell immune response, Delayed-type hypersensitivity, American cutaneous leishmaniasis

## Abstract

**Background:**

American cutaneous leishmaniasis (ACL) is a protozoan parasitic disease caused by different *Leishmania* spp. from *L.* (*Leishmania*) and *L.* (*Viannia*) subgenera. In Brazil, seven *Leishmania* spp. act as ACL agents. Infection with *L.* (*L.*) *amazonensis* presents a wide clinical–immunopathological spectrum, ranging from localized cutaneous leishmaniasis (LCL), which usually responds well to antimony therapy, to borderline disseminated cutaneous leishmaniasis (BDCL), which may require twice as much LCL-antimony therapy to cure, and, finally, to anergic diffuse cutaneous leishmaniasis (ADCL), which is highly resistant to any chemotherapy. This clinical variability is driven by different degrees of T-cell immunosuppression, which negatively impact delayed-type hypersensitivity (DTH), as assessed by the Montenegro skin test (MST).

**Methods:**

Given MST’s role as a T-cell-mediated resistance marker, we used it for diagnosing and monitoring patients with LCL (*n* = 8) and BDCL (*n* = 3) due to *L.* (*L.*) *amazonensis* to assess T-cell immunosuppression in these patients. MST was assessed at diagnosis and after treatment, although one patient with LCL refused treatment. The study took place at the Evandro Chagas Institute (Pará, Brazil), with diagnosis confirmed through parasitological assays, MST using *L.* (*V.*) *braziliensis* antigen, indirect fluorescent antibody test (IFAT)-immunoglobulin (Ig)G serology, histopathology, and PCR. Phenotypic and genotypic analyses were used to identify the primary agent.

**Results:**

MST was negative in all patients, both before and after successful treatment. Notably, one patient with LCL who declined treatment also showed no MST reactivity both before and after spontaneous healing. The absence of MST reactivity persisted for up to 1 year postcure in LCL and up to 2 years in BDCL, indicating a sustained lack of MST reactivity regardless of treatment outcome or spontaneous healing.

**Conclusions:**

The lack of MST reactivity pre- and post-treatment in LCL and BDCL challenges the role of DTH as a marker for T-cell-mediated resistance to *L.* (*L.*) *amazonensis* infection. Moreover, the first case of spontaneous LCL cure by *L.* (*L.*) *amazonensis* showed no MST reactivity pre- or post-resolution, raising doubts about the role of DTH in this process.

**Graphical Abstract:**

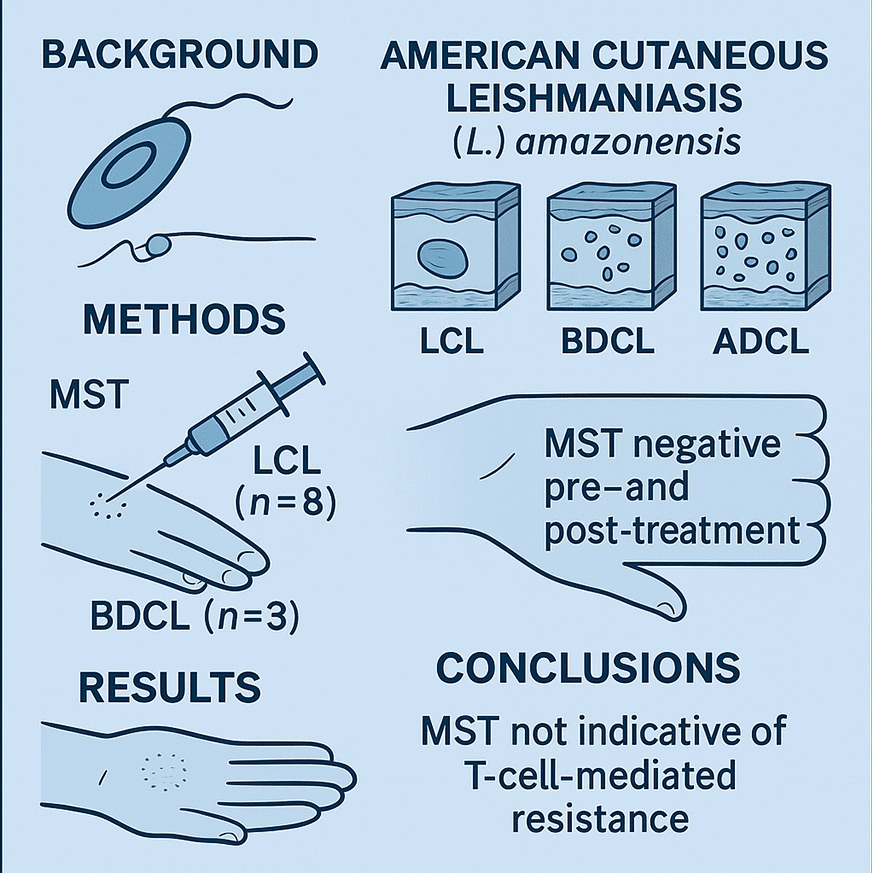

**Supplementary Information:**

The online version contains supplementary material available at 10.1186/s13071-025-06941-6.

## Background

American cutaneous leishmaniasis (ACL) is an infectious parasitic disease caused by at least 15 distinct protozoan parasites of the genus *Leishmania*. These leishmanine parasites are taxonomically classified into three subgenera: *L.* (*Leishmania*), *L.* (*Viannia*), and *L.* (*Mundinia*). Currently, ACL is regarded as one of the most complex parasitic diseases due to the multiplicity of interactions between the wide heterogeneity of *Leishmania* species and the human immune response [1–3].

In Brazil, seven *Leishmania* spp. and a hybrid parasite are linked to ACL [4, 5], with *L.* (*V.*) *braziliensis* and *L.* (*L.*) *amazonensis* being particularly significant from a medical perspective. *Leishmania* (*V.*) *braziliensis*, the first pathogenic *Leishmania* species identified in the Americas, is widely distributed across Central and South America, including all geographic regions of Brazil [6]. It is associated with a broad clinical spectrum, including localized cutaneous leishmaniasis (LCL), borderline disseminated cutaneous leishmaniasis (BDCL), and mucocutaneous leishmaniasis (MCL), the latter of which causes severe inflammatory responses and tissue damage in the nasobuccopharyngeal mucosa.

*Leishmania* (*L.*) *amazonensis*, the second pathogenic *Leishmania* species identified in Brazil, is of significant medical relevance, particularly in the Brazilian Amazon, where it plays a key role in the epidemiology of ACL [7]. It also contributes to a broad clinical–immunopathological spectrum, including both LCL and BDCL, as well as anergic diffuse cutaneous leishmaniasis (ADCL), a rare but severe form characterized by extensive, diffusely distributed nodular lesions. ADCL is highly mutilating and remains incurable, further underscoring the clinical importance of *L.* (*L.*) *amazonensis* in the clinical–immunopathological spectrum of ACL [8–10].

*Leishmania* (*V.*) *braziliensis* and *L.* (*L.*) *amazonensis* show distinct immunopathological profiles, underscoring their differential interactions with the human immune system. *Leishmania* (*V.*) *braziliensis* triggers a strong proinflammatory response characterized by a CD4⁺/Th1-type immune response, with increased interferon gamma (IFN-γ) and tumor necrosis factor alpha (TNF-α) levels, which contribute to the severe tissue damage in MCL. In contrast, *L.* (*L.*) *amazonensis* induces a pronounced anti-inflammatory response driven by a CD4⁺/Th2-type immune response, characterized by high levels of interleukin (IL)-10 and transforming growth factor (TGF)-β, which underlies the pathogenesis of ADCL. These species play opposing roles in immunomodulating ACL pathogenesis, showing a strong species-specific influence on the clinical–immunopathological spectrum of the disease [11, 12].

Current research on the human immune response in ACL emphasizes the need for further investigation into delayed-type hypersensitivity (DTH), which is closely associated with a protective CD4⁺/Th1-type immune response. DTH is routinely assessed through the Montenegro skin test (MST). Since the 1980s, several studies conducted at the Ralph Lainson Leishmaniasis Laboratory (RL/LL) of the Evandro Chagas Institute in Pará, Brazil, have focused on DTH suppression in *L.* (*L.*) *amazonensis*-induced ACL. Notably, 57.7% patients with LCL infected with *L.* (*L.*) *amazonensis* showed no MST reactivity, regardless of disease duration, suggesting that the lack of MST reactivity is due to significant DTH suppression by the parasite rather than a transient MST failure [13].

Subsequent studies have reinforced the lack of MST reactivity in LCL due to *L.* (*L.*) *amazonensis*, with reported rates ranging from 48.6% to 100% [8, 14–16]. While LCL generally responds well to antimony therapy, MST negativity has also been observed at 100% in BDCL and ADCL, both of which have higher parasitic loads. BDCL requires double the antimony dose used for LCL, while ADCL is highly resistant to chemotherapy. These findings strongly suggest that *L.* (*L.*) *amazonensis* infection systematically suppresses the T-cell immune response, particularly the CD4⁺/Th1-type immune response, impairing DTH expression and MST reactivity across the clinical–immunopathological spectrum of ACL [9, 10, 12, 17, 18].

Supporting these findings, lymphocyte proliferative responses in patients with LCL and ADCL caused by *L.* (*L.*) *amazonensis* were significantly lower compared with those in patients with LCL and patients with ML caused by *L.* (*V.*) *braziliensis*. This difference was noted when lymphocytes were stimulated with crude or recombinant *Leishmania* spp. antigens, further highlighting the suppressive effect of *L.* (*L.*) *amazonensis* on the host T-cell immune response [19].

A recent study reinforces the systematic suppression of *L.* (*L.*) *amazonensis* on the human T-cell immune response, particularly its negative impact on DTH as assessed by MST. This suppression was evident at the time of diagnosis in eight patients with LCL and three patients with BDCL and persisted even after antimony therapy. Notably, one patient with LCL who declined treatment recovered spontaneously within a year. MST negativity was observed 3 months to 1 year postcure in LCL cases and 1–2 years postcure in BDCL cases. While previously reported in LCL [14], this event in BDCL represents a novel finding. These results challenge the reliability of DTH as a marker of (CD4⁺/Th1-mediated) immune resistance against *L.* (*L.*) *amazonensis* infection.

Currently, we aim to investigate the significance of these findings in ACL due to *L.* (*L.*) *amazonensis*, particularly in patients with LCL and patients with BDCL. The overarching goal is to deepen the understanding of these immunomodulatory mechanisms, thereby optimizing clinical management and therapy strategies for affected patients.

## Methods

### Patients and procedures

All patients (eight with LCL and three with BDCL) included in this study were seen at the outpatient clinic of the Ralph Lainson Leishmaniasis Laboratory (RL/LL), Parasitology Department of the Evandro Chagas Institute (Ministry of Health), Pará, Brazil, at the time between 2016 and 2023. They underwent routine procedures for the laboratory diagnosis of ACL, including direct parasitological analysis, culture on Difco B45 medium for parasite isolation, MST using *L. *(*V.*) *braziliensis* promastigote antigen, indirect fluorescent antibody test (IFAT)-immunoglobulin (Ig)G serology with *L. *(*L.*) *amazonensis* axenic amastigote antigen, histopathology, and PCR. Isolated parasite samples were phenotypic and/or genotypic characterized following standardized analysis [4, 20].

### Study design

This prospective study analyzed clinical and laboratory data from patients with suspected ACL who attended the RL/LL outpatient clinic between 2016 and 2023. The primary objective was to identify cases of *L. *(*L.*) *amazonensis*-induced ACL, particularly LCL and BDCL, in which patients exhibited negative MST results at diagnosis but achieved clinical cure following antimony therapy. The secondary objective was to reassess these patients posthealing with a new MST to evaluate their capacity for MST conversion. Since MST reactivity serves as a direct indicator of DTH expression, which is associated with the protective CD4⁺/Th1-type immune response, its conversion is regarded as key immunological marker of resistance to *Leishmania* infection [21].

### Clinical features of the patients

The clinical features of patients with LCL and patients with BDCL were in alignment with both our prior clinical experience and the Tegumentary Leishmaniasis Surveillance Manual (Ministry of Health, Brazil) [8, 15, 17, 22, 23]. The key clinical features, including the morphology, number, and location of skin lesions for each clinical form, as well as the duration of disease progression, will be summarized in the following section and illustrated in Supplementary Material Figs. 1 and 2 and in a table as well.

### Antimonial regimen for treating patients

All patients were prescribed antimony therapy at a dosage of 12 mg Sb^v^/kg/body weight; however, one patient with LCL declined the treatment. The seven patients with LCL underwent two 22-day cycles, separated by a 10-day interval, while the three patients with BDCL received three to four cycles. These antimony regimens, in use for over two decades, have yielded satisfactory therapeutic outcomes for both LCL and BDCL, regardless of whether the causative agent was *L.* (*L.*) *amazonensis* or *L.* (*V.*) spp. [8, 14, 15, 17].

### Montenegro skin test (MST)

MST was inserted at the RL/LL nearly 50 years ago using *L. *(*L.*) *amazonensis* promastigote antigen for the diagnosis of ACL [24]. It kept effective until the late 1990s, when lymphocyte proliferation assays showed that the *L. *(*V.*) *braziliensis* antigen induced significantly higher responses than the *L. *(*L.*) *amazonensis* antigen [19]. As a result, *L. *(*V.*) *braziliensis* replaced *L. *(*L.*) *amazonensis* in MST, improving reactivity and specificity, as *L. *(*Viannia*) spp. are the primary ACL agents in the region [8]. Despite using *L. *(*V.*) *braziliensis* antigen in MST, previous studies documented DTH suppression in ACL due to *L. *(*L.*) *amazonensis*, even with a homologous antigen [13, 14]. This strongly suggests that DTH suppression is not specific to the antigen used in MST but is instead an immunological event specifically associated with *L. *(*L.*) *amazonensis* infection [3, 9, 10].

## Results

### Patients

Based on the clinical and laboratory criteria of this study, 11 male patients with ACL infected with *L. *(*L.*) *amazonensis*, eight with LCL, and three with BDCL, with mean ages of 44.2 and 35.3 years, respectively, were selected for MST evaluation following clinical cure with antimony therapy. The limited sample size was primarily due to the low prevalence of *L. *(*L.*) *amazonensis*-induced ACL, accounting for less than 2% of ACL cases due to *L. *(*Viannia*) spp., and patient refusal to participate. Despite these restrictions, the results were welcome.

### Clinical features

The clinical features in the eight patients with LCL was marked by one to three nodular-ulcerated skin lesions, primarily located on the upper or lower limbs; however, one case presented a single lesion in the frontal region of the head (Supplementary Material Fig. 1). The lesions had an evolution period of 2–6 months. By contrast, the three BDCL patients presented with a significantly higher number of lesions, ranging from 4 to 12, mainly affecting the upper limbs but also extending to the lower limbs and trunk. These lesions were primarily infiltrated plaques and had a longer duration, lasting 6–18 months (Supplementary Material Fig. 2). Additionally, Table 1 presents the personal and clinical data of all patients included in the study, encompassing both LCL and BDCL cases (Table 1).

**Table 1:** Personal and clinical characteristics of patients with LCL and BDCL caused by *Leishmania *(*L.*) *amazonensis* included in this study

Clinical Forms	Patients (*n*)	Gender	Mean age*	Time of disease**	Lesion number	Lesion traits
LCL	8	Male	44.2	2–6	1–3	Nodular-ulcerated
BDCL	3	Male	35.3	6–18	4–12	Infiltrated-plaques

^*^Years

^**^Months

*LCL* localized cutaneous leishmaniasis,

*BDCL* borderline disseminated cutaneous leishmaniasis

### Laboratory diagnosis

*Leishmania* was detected in skin lesions smears and culture medium for both patients with LCL and patients with BDCL. All isolated parasites samples were identified as *L. *(*L.*) *amazonensis*. Serological analysis showed a positive IFAT-IgG response in all patients, with LCL cases showing low-to-moderate titers (80–320) and BDCL cases moderate-to-high titers (640–1,250). Histopathology revealed a dermal mononuclear cell infiltrate dominated by vacuolated macrophages, which were heavily parasitized in BDCL, along with a few plasma cells and lymphocytes.

The absence of necrotic areas revealed a typical vacuolated macrophage reaction in the dermis (Supplementary Material Fig. 3).

### Antimony therapy

Antimony treatment regimen (12 mg Sb^v^/kg/body weight intravenously once daily for 22 days) was used in two series with a 10-day interval between them to seven patients with LCL (one patient declined treatment). For the three patients with BDCL, the regimen consisted of three series for one patient and four series for two patients, with a 10-day interval between the first two series and a 15-day interval before the final series. This treatment resulted in clinical cure within an average of 2 months for patients with LCL and 4.5 months for patients with BDCL, with no significant adverse effects requiring treatment interruption.

One of the eight patients with LCL, a 42-year-old man with a single lesion on the right shoulder, declined antimony therapy, leading to spontaneous disease progression for up to one year. Upon reevaluation, he agreed to a new clinical and laboratory assessment, including a repeat MST. By this time, his lesion had completely regressed and healed, representing the first documented case of spontaneous cure in an patient with LCL due to *L. *(*L.*) *amazonensis*. Notably, the patient tested negative on the MST both during active infection and after the spontaneous resolution of the disease.

### MST evaluation

This case of spontaneous healing in an patient with LCL due to *L. *(*L.*) *amazonensis* provided a unique opportunity to further investigate DTH responses. Notably, MST results remained negative in all patients following clinical cure, either after antimony therapy (seven LCL and three BDCL) or spontaneously (one LCL). These findings confirm the sustained absence of DTH expression in both patients with LCL and patients with BDCL with *L. *(*L.*) *amazonensis* infection, persisting beyond active disease and clinical resolution, regardless of the mode of cure.

## Discussion

Since its development in 1926, MST has served as a critical diagnostic tool for cutaneous leishmaniasis (CL) globally, particularly for evaluating DTH to *Leishmania* infection [25]. It remains widely used in Latin America, where the principal etiological agents of ACL belong to the *L. *(*Viannia*) subgenus, including *L. *(*V.*) *braziliensis*, *L. *(*V.*) *peruviana*, *L. *(*V.*) *panamensis*, and *L. *(*V.*) *guyanensis*, which are well-recognized for their ability to stimulate a CD4⁺/Th1-type immune response and thereby elicit DTH reactions [26–29]. Recently, MST has been employed not only for its original diagnostic purpose but also for epidemiological studies as a screening tool for DTH responses associated with asymptomatic infections caused by *L.* (*L.*) *infantum* and *L.* (*L.*) *donovani* in regions endemic for visceral leishmaniasis. These applications further support the ability of MST as a reliable method for assessing DTH in human *Leishmania* infections [30–41].

By contrast, early studies into the use of MST in cases of ACL caused by *L. *(*L.*) *amazonensis* or *L.* (*L.*) *pifanoi*, particularly in ADCL form, were conducted several decades ago in Brazil [42, 43], Bolivia [44], and Venezuela [45]. These studies consistently reported negative MST results in patients with ADCL, leading to the prevailing view that this clinical form is linked to a specific immunodeficiency in the host. As a result, the absence of DTH in ADCL has been largely attributed to the host’s inability to generate an effective T-cell-mediated immune response, rather than to any direct immunosuppressive action of the parasite itself [46].

DTH suppression due to *L. *(*L.*) *amazonensis* infection was first detected in Brazil during the 1980s through a pioneering study conducted by our research group in Pará State. This study was the first to demonstrate that 57.7% of patients with LCL caused by *L. *(*L.*) *amazonensis* showed no MST reactivity when using homologous promastigote antigen [13]. Subsequent studies confirmed comparable or even higher rates of DTH suppression, ranging from 48.6% to 100% among patients with LCL, thereby establishing the absence of MST reactivity as a hallmark of *L. *(*L.*) *amazonensis* infection across the clinical–immunopathological spectrum of ACL. This phenomenon was most pronounced in ADCL, where MST reactivity was universally absent (100%), but was also observed to varying degrees in patients with LCL (48.6–100%), despite their generally favorable response to antimony therapy [8, 9, 14–16].

These findings of DTH suppression in *L. *(*L.*) *amazonensis*-induced LCL is further supported by histopathological findings. Skin lesions typically exhibit a dermal cellular infiltrate that is predominantly macrophagic, with scarce plasma cells and lymphocytes, and a notably rare presence of epithelioid cells, i.e., macrophages that are activated through T-cell-mediated immune response. The macrophages appear vacuolated and heavily parasitized, providing additional confirmation of impaired DTH expression at the site of infection [47]. This histological profile contrasts sharply with that observed in *L.* (*Viannia*) *braziliensis*-induced LCL, where the dermal infiltrate is characterized by a prominent presence of epithelioid cells, corresponding with a high prevalence (≥ 90%) of MST reactivity [48].

The present results demonstrated a complete absence of MST reactivity in all patients analyzed, including those with LCL and BDCL, irrespective of lesion count, clinical presentation, or disease duration, both before and after successfully antimony therapy. Although the absence of MST reactivity in LCL caused by *L. *(*L.*) *amazonensis* has been previously documented [14], this is the first report of such a finding in BDCL. Notably, in contrast to *L. *(*V.*) *braziliensis* infection, in which MST reactivity persists over time, even following antimony therapy, due to continued antigenic stimulation [49, 50], infection with *L. *(*L.*) *amazonensis* in both LCL and BDCL appears to be completely cleared by therapy. This clearance likely prevents the sustained antigenic stimulation of the T-cell-mediated immune response, including DTH, as assessed by MST. These findings suggest a previously unrecognized immunological profile linked to *L. *(*L.*) *amazonensis* infection and highlight the distinct immune mechanisms involved in ACL pathogenesis.

Of particular note is the first documented case of LCL spontaneous cure due to *L. *(*L.*) *amazonensis*, observed 1 year after diagnosis. Remarkably, the patient exhibited no MST reactivity either before or after the spontaneous resolution of the lesion, an observation that challenges current understanding of DTH in human *Leishmania* infection [26–29, 51]. Typically, spontaneous cure is associated with the activation of the T-cell-mediated immune response. However, this case suggests that *L. *(*L.*) *amazonensis* infection may inhibit the activation of genetic pathways essential for DTH expression, most likely linked to the CD4⁺/Th1-type immune response, thereby compromising macrophage-mediated resistance to infection [21, 52].

These findings raise a critical question: why do patients with *L. *(*L.*) *amazonensis*-induced LCL and BDCL, despite achieving clinical cure, whether through antimony therapy or spontaneously, fail to develop DTH, assessed by MST reactivity? This contrasts with patients affected by so-called Old and New World visceral leishmaniasis, who, despite profound suppression of the CD4⁺/Th1-type immune response, often exhibit a significant shift toward DTH, assessed by MST reactivity following treatment [53, 54]. A plausible explanation lies in the persistence of low-level infection. In *L. *(*V.*) *braziliensis*-induced LCL, for instance, antimony therapy does not fully eradicate the parasite from healed lesions, allowing ongoing antigenic stimulation and sustained DTH responses [49, 50]. Similarly, in treated or asymptomatic visceral leishmaniasis, incomplete parasite clearance, particularly in the liver, may promote posttreatment DTH conversion through the gradual reactivation of CD4⁺/Th1-type immune response [53–56]. By contrast, *L. *(*L.*) *amazonensis* infection appears to be entirely eliminated by treatment, potentially precluding such immunological reactivation.

In this case, DTH suppression in *L. *(*L.*) *amazonensis* infection, both in LCL and BDCL, appears to be influenced by two distinct parasite–host interaction mechanisms. The first involves the potential inhibition of genetic pathways that regulate DTH during the active phase of infection [21, 52]. The second, which may represent a novel observation, is associated with the absence of antigenic stimulation following the complete clearance of the parasite through antimony therapy. These findings underscore the critical role of species-specific host–parasite interactions in modulating the T-cell-mediated immune response, particularly the CD4⁺/Th1 and CD4⁺/Th2 pathways, which in turn govern DTH activation [3, 8, 9].

Supporting this hypothesis, it is noteworthy that, based on more than 40 years of clinical experience monitoring patients infected with *L. *(*L.*) *amazonensis* in Pará, Brazilian Amazon, this research group has not observed any cases of relapse following medium- or long-term cure with antimony therapy, particularly among patients with LCL. This observation stands in contrast to the more frequent relapses seen in LCL and BDCL cases due to *L. *(*V.*) *braziliensis* or *L.* (*V.*) *guyanensis* [Silveira, personal observation].

The progression of *L. *(*L.*) *amazonensis* infection appears to be influenced by the extent of DTH suppression, mainly due to the impairment of the CD4⁺/Th1-type immune response, in conjunction with host-specific factors such as age and genetic background. This may lead to the development of BDCL, an intermediate-severity form that predominantly affects young adults and typically requires twice the standard dosage of antimony therapy used for LCL [8, 9, 17]. In the absence of effective immune control, the infection may progress to ADCL, a severe and incurable form resistant to all types of chemotherapy. ADCL primarily affects children under 10 years of age and adults belonging to ethnic groups with heightened susceptibility to *L. *(*L.*) *amazonensis* infection [2, 10]. This clinical form is most frequently observed among historically enslaved individuals of African descent in the pre-Amazon region of Maranhão State, which reports the highest number of ADCL cases in Brazil [57].

In the majority of infected individuals, particularly those with LCL, accounting for ≥ 98% of cases, the parasite’s suppressive effect on the CD4⁺/Th1-type immune response, reflected by the absence of DTH in negative MST, appears to be less pronounced than in patients with BDCL and patients with ADCL. This relative preservation of immune function may be alternatively attributed to the immunomodulatory activity of CD8⁺ T cells, which exhibit significantly higher densities (*P* < 0.05) than CD4⁺ T cells across the clinical–immunopathological spectrum of ACL due to *L. *(*L.*) *amazonensis*, even after antimony therapy [12, 58]. These findings highlight the pivotal role of CD8⁺ T cells as a key resistance mechanism in the control of *L. *(*L.*) *amazonensis* infection, contributing to protective immunity by producing IFN-γ and promoting Th1 responses not only in human infection [8, 9, 12] but also in murine models [59, 60].

*Leishmania *(*L.*) *amazonensis* primarily modulates the human immune response by disrupting the balance between cellular and humoral immunity. Specifically, it suppresses the CD4⁺/Th1-type immune response, including DTH, which is essential for macrophage activation and effective parasite clearance, while enhancing the less effective, IgG-mediated humoral response [61, 62]. This immunomodulatory effect is particularly pronounced in BDCL and ADCL, which are characterized by high parasite loads [3, 8, 9, 63]. In the present study, patients with LCL exhibited low to moderate IgG titers (80–320), whereas those with BDCL showed significantly higher titers (640–2,560). These findings support the notion that *L. *(*L.*) *amazonensis* facilitates disease progression by impairing protective cellular immunity, primarily the CD4⁺/Th1-type response, while promoting a nonprotective humoral response.

Supporting these observations, a recent methodological advancement deserves attention: the optimization of MST using an *L.* (*V.*) *lainsoni* axenic amastigote antigen to assess DTH in ACL cases in the Brazilian Amazon. This approach yielded significantly higher mean reactivity (18.8 mm ± 13.3) in LCL caused by *L. *(*Viannia*) species, compared with the response elicited by the conventional *L. *(*V.*) *braziliensis* promastigote antigen (11.8 mm ± 8.2). Notably, the only patient in the study diagnosed with LCL due to *L. *(*L.*) *amazonensis* exhibited no reactivity to the MST, in contrast to 52 cases associated with *L. *(*Viannia*) species. This finding reinforces the suppression of DTH in *L. *(*L.*) *amazonensis* infections, even when using an antigen demonstrated to be more potent than the standard *L. *(*V.*) *braziliensis* antigen [64].

At the time of concluding this study, two individuals previously diagnosed with *L. *(*L.*) *amazonensis*-associated ACL were reevaluated. The first was a 58-year-old woman with ADCL, and the second was a 54-year-old man with BDCL. Both had undergone multiple treatment regimens, including antimonial compounds, pentamidine, and chemoimmunotherapy (BCG combined with Leishvacin), and they ultimately achieved clinical cure (Fig. 4, supplementary material) [17, 65]. Immunological testing revealed negative IFAT/enzyme-linked immunosorbent assay (ELISA)-IgG results for the woman and a negative MST for the man, confirming parasitological cure. Notably, the man’s prior MST conversion, documented 25 years earlier, was likely attributable to transient immunogenic stimulation induced by immunotherapy. These findings represent the first documented confirmation of parasitological cure in ADCL and BDCL caused by *L. *(*L.*) *amazonensis*, as evidenced by negative serological and DTH-based assays.

Finally, to underscore the complexity of the immune responses to *L. *(*L.*) *amazonensis* infection, it should be highlighted that an ADCL case with more than 30 years of disease evolution achieved clinical cure following a single intranasal dose of meglumine antimoniate, despite the patient remaining negative for the MST after cure [66]. Notably, this therapeutic approach was unsuccessful in four patients under our clinical supervision [67].

## Conclusions

This study unequivocally demonstrated that *L. *(*L.*) *amazonensis* infection significantly suppresses the human T-cell immune response, affecting both CD4⁺/Th1-type activity and DTH. The consistent absence of MST reactivity in patients with LCL and patients with BDCL, before and after antimony therapy, challenges the reliability of DTH as an indicator of T-cell-mediated resistance to this infection.

## Supplementary Information

Additional file 1. Figure 1. *(A)* LCL case due to *L. *(*L.*) *amazonensis*, presenting an ulcerated, infiltrated lesion with a thickened border, located on the right frontal region of the head, with a two-month evolution. *(B)* The same case following two courses of antimony therapy, showing complete regression of the infiltrative process at the lesion’s border and evidence of skin healing.

Additional file 2. Figure 2. *(A)* BDCL case due to *L. *(*L.*) *amazonensis*, showing a primary infiltrated plaque on the right shoulder with disseminated papulo-nodular lesions extending to the arm. *(B)* The same case after three courses of antimony therapy, significant regression of both primary and secondary lesions was observed.

Additional file 3. Figure 3. *(A)* Histological section of a LCL skin lesion due to *L. *(*L.*) *amazonensis*, showing a dermal mononuclear infiltrate composed predominantly of vacuolated macrophages containing parasites (arrows), along with lymphocytes and plasma cells. *(B)* Histological section of a BDCL skin lesion due to *L. *(*L.*) *amazonensis*, demonstrating a similar dermal mononuclear infiltrate with heavily parasitized vacuolated macrophages (arrows), accompanied by lymphocytes and plasma cells. *Eosin x hematoxylin staining*; *bars* = *20 µm*.

Additional file 4. Figure 4. Right column - A: BDCL case due to *L. *(*L.*) *amazonensis*, presenting with nodular-infiltrated lesions on both ears, the right hemiface, and the nasal wing, along with a larger infiltrated plaque on the dorsal right hand and wrist, evolving over one year and six months. A 7-year-old girl with ADCL evolving three years ago, exhibiting diffuse nodular cutaneous lesions on her legs and arms. By age 29, the disease persisted with increased cutaneous infiltration, nodules, and papules, when a chemo-immunotherapy regimen was administered. Left column - B: Both cases cured two years after a chemo-immunotherapy regime (pentamidine plus BCG+Leishvacin).

## Data Availability

Data supporting the main conclusions of this study are included in the manuscript.

## Abbreviations

ACL: American cutaneous leishmaniasis
MST: Montenegro skin test
DTH: Delayed-type hypersensitivity response
LCL: Localized cutaneous leishmaniasis
BDCL: Borderline disseminated cutaneous leishmaniasis
ML: Mucosal leishmaniasis
ADCL: Anergic diffuse cutaneous leishmaniasis
PCR: Polymerase chain reaction
RL/LL: Ralph Lainson Leishmaniases Laboratory
IFAT: Indirect fluorescent antibody test
IgG: Immunoglobulin G
RFLP: Restriction fragment length polymorphism
RNA: Ribonucleic acid
CD4/Th1: Lymphocyte CD4/T-helper type 1
CD4/Th2: Lymphocyte CD4/T-helper type 2
