# Supplementary figures and images for: Systematic suppression of Leishmania (Leishmania) amazonensis-mediated delayed-type hypersensitivity response in American cutaneous leishmaniasis
Source: Parasit Vectors. 2025 Aug 5;18:336. doi: 10.1186/s13071-025-06941-6 (PMC12326837; doi:10.1186/s13071-025-06941-6)

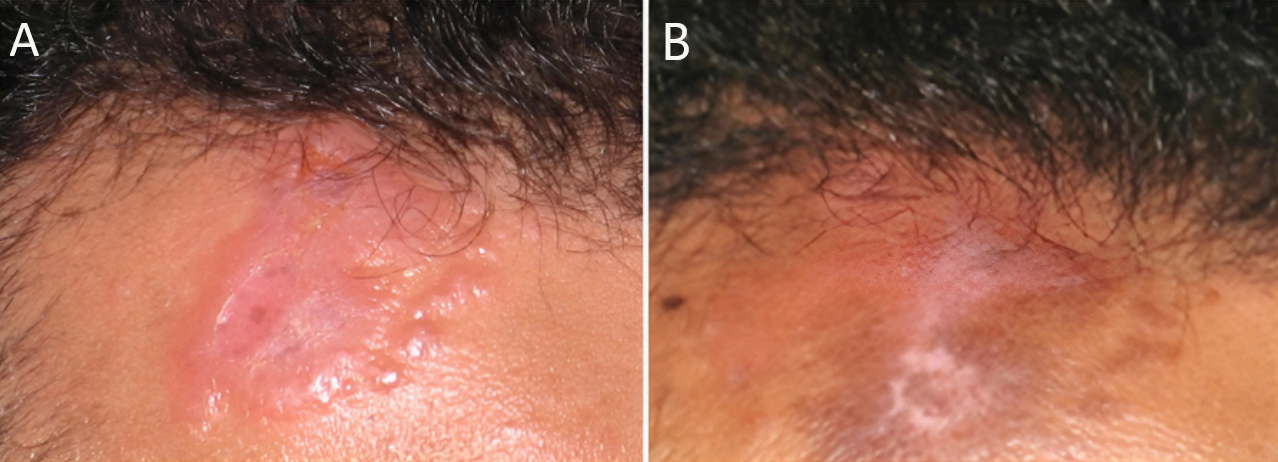

Supplement: Supplementary file 1 — Additional file 1. Figure 1. (A) LCL case due to L. (L.) amazonensis, presenting an ulcerated, infiltrated lesion with a thickened border, located on the right frontal region of the head, with a two-month evolution. (B) The same case following two courses of antimony therapy, showing complete regression of the infiltrative process at the lesion’s border and evidence of skin healing. [file 13071_2025_6941_MOESM1_ESM.tif]

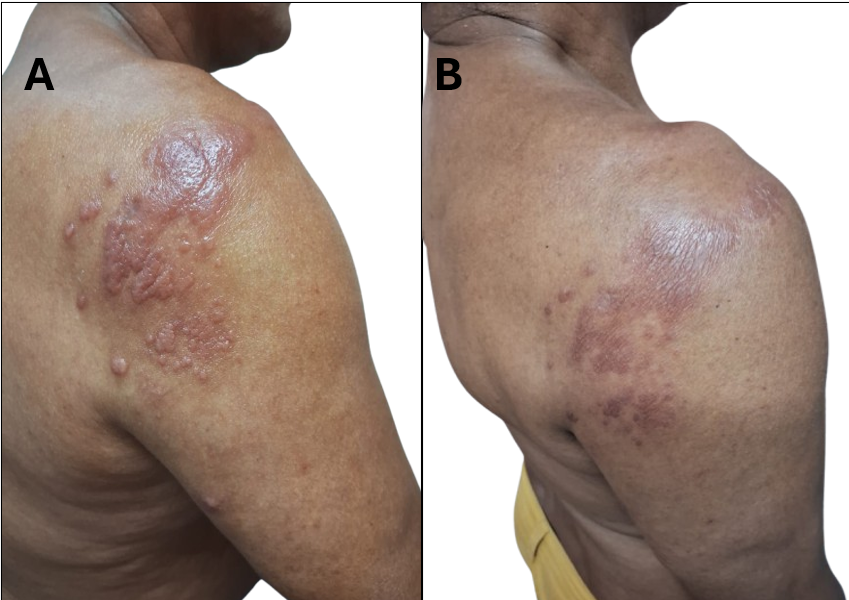

Supplement: Supplementary file 2 — Additional file 2. Figure 2. (A) BDCL case due to L. (L.) amazonensis, showing a primary infiltrated plaque on the right shoulder with disseminated papulo-nodular lesions extending to the arm. (B) The same case after three courses of antimony therapy, significant regression of both primary and secondary lesions was observed. [file 13071_2025_6941_MOESM2_ESM.tif]

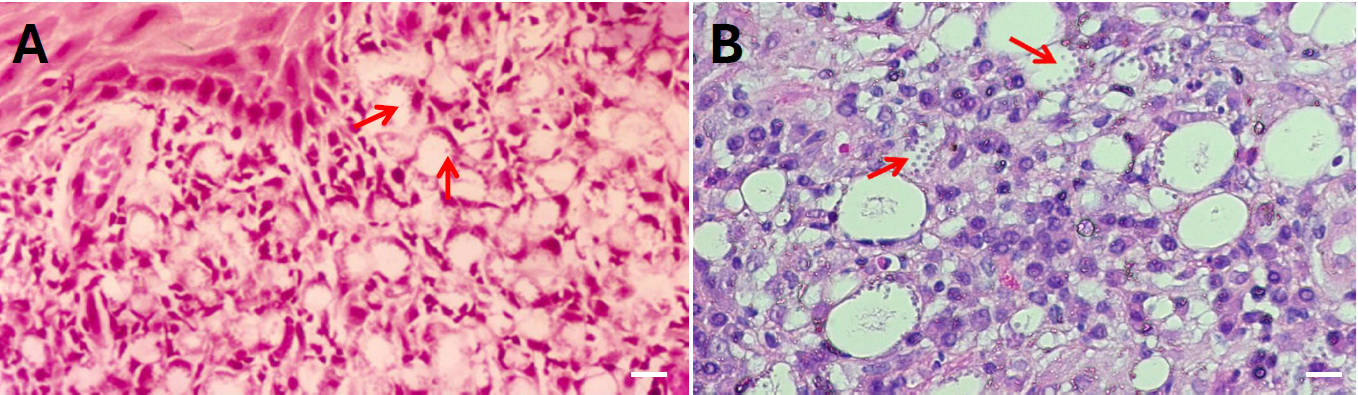

Supplement: Supplementary file 3 — Additional file 3. Figure 3. (A) Histological section of a LCL skin lesion due to L. (L.) amazonensis, showing a dermal mononuclear infiltrate composed predominantly of vacuolated macrophages containing parasites (arrows), along with lymphocytes and plasma cells. (B) Histological section of a BDCL skin lesion due to L. (L.) amazonensis, demonstrating a similar dermal mononuclear infiltrate with heavily parasitized vacuolated macrophages (arrows), accompanied by lymphocytes and plasma cells. Eosin x hematoxylin staining; bars = 20 µm. [file 13071_2025_6941_MOESM3_ESM.tif]

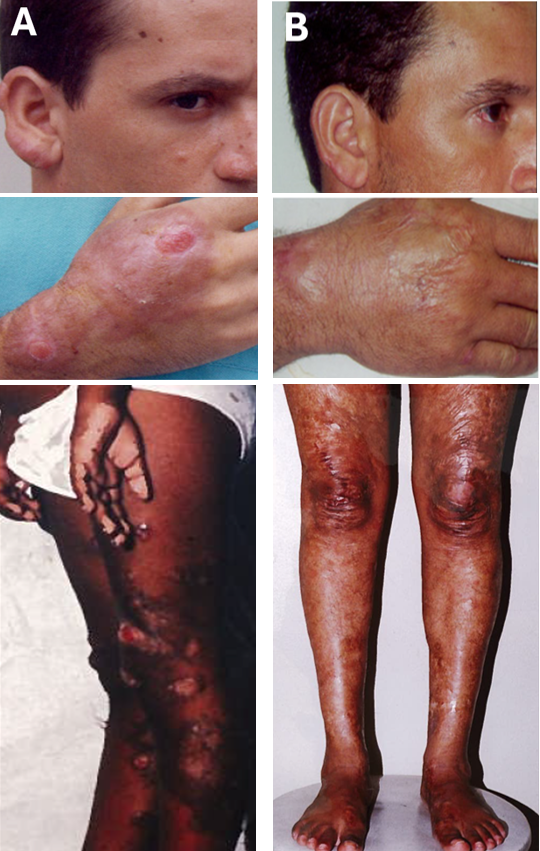

Supplement: Supplementary file 4 — Additional file 4. Figure 4. Right column - A: BDCL case due to L. (L.) amazonensis, presenting with nodular-infiltrated lesions on both ears, the right hemiface, and the nasal wing, along with a larger infiltrated plaque on the dorsal right hand and wrist, evolving over one year and six months. A 7-year-old girl with ADCL evolving three years ago, exhibiting diffuse nodular cutaneous lesions on her legs and arms. By age 29, the disease persisted with increased cutaneous infiltration, nodules, and papules, when a chemo-immunotherapy regimen was administered. Left column - B: Both cases cured two years after a chemo-immunotherapy regime (pentamidine plus BCG+Leishvacin). [file 13071_2025_6941_MOESM4_ESM.tif]
